# Trends in Reported Sexual Behavior and Y-Chromosomal DNA Detection Among Female Sex Workers in the Senegal Preexposure Prophylaxis Demonstration Project

**DOI:** 10.1097/OLQ.0000000000001175

**Published:** 2020-03-16

**Authors:** D. Allen Roberts, Stephen E. Hawes, Mame D. Bousso Bao, Anna Julienne Ndiaye, Daouda Gueye, Dana N. Raugi, Moustapha Mane, Aminata Mboup, Ousmane Diouf, Fatima Jones, Coumba Toure Kane, Moussa Sarr, Souleymane Mboup, Geoffrey S. Gottlieb

**Affiliations:** From the Departments of ∗ Epidemiology; † Global Health; ‡ Health Services, University of Washington, Seattle, WA; § IRESSEF: Institut de Recherche en Santé de Surveillance Epidemiologique et de Formations, Pole Urbain de Diamniadio, Dakar, Senegal; ¶ Department of Medicine, Allergy and Infectious Diseases, Center for Emerging and Re-Emerging Infectious Diseases, University of Washington, Seattle, WA; ∥ Westat, Inc., Rockville, MD

## Abstract

**Background:**

Preexposure prophylaxis (PrEP) can reduce HIV acquisition among female sex workers (FSWs). However, changes in condomless sex frequency after PrEP initiation could reduce PrEP effectiveness when PrEP adherence is suboptimal as well as increase the risk of acquiring other sexually transmitted infections. Objective measures of condomless sex may be more accurate for determining changes in sexual behavior than self-reported measures.

**Methods:**

We longitudinally measured self-reported condom use, number of clients, and presence of Y-chromosomal DNA (Yc-DNA) in vaginal swabs among 267 FSWs accessing PrEP at 4 clinics in Senegal between 2015 and 2016. We assessed trends in sexual behavior over time since PrEP initiation using generalized estimating equations and evaluated predictors of Yc-DNA detection.

**Results:**

We found no increase in self-reported condomless sex with clients (odds ratio [OR], 0.94; 95% confidence interval [CI], 0.89–1.00), main partners (OR, 0.99; 95% CI, 0.96–1.02), or Yc-DNA detection (OR, 0.99; 95% CI, 0.90–1.08) over time since initiation. Y-chromosomal DNA was detected in 34 (22%) of 154 swabs tested and in 15 (26%) of 58 swabs from FSW reporting consistent condom use among both clients and main partners. Self-reported condom use with clients or main partners did not predict Yc-DNA detection.

**Conclusions:**

In a FSW PrEP demonstration project in Senegal, we found no evidence of risk compensation among FSWs on PrEP as measured by self-reported behavior or through Yc-DNA detection. Y-chromosomal DNA detection was frequently detected among FSWs reporting consistent condom use, highlighting limitations of self-reported sexual behavioral measures.

## Abstract 2

A study among female sex workers in Senegal found no evidence of increased sexual risk behavior after preexposure prophylaxis initiation as measured by self-report or Y-chromosomal DNA detection.

Supplemental digital content is available in the text.

Despite increased access to HIV treatment and prevention options, an estimated 1.8 million people globally became newly infected with HIV in 2017.^1^ Key populations are disproportionately affected by HIV and account for nearly half of new infections.^1^ World Health Organization guidelines recommend offering preexposure prophylaxis (PrEP) to individuals at substantial risk of HIV infection, including female sex workers (FSWs).^2^ Despite the potential benefits of PrEP use among FSWs, condom use is still recommended while taking PrEP because of concerns about adherence to daily medication, sexually transmitted infection (STI) acquisition, and pregnancy.^3^ Female sex workers who benefit from PrEP may be incentivized to reduce condom use secondary to condom availability or costs, client preference, ability to charge more for condomless sex, and concerns about client violence.^4,5^ If clients are aware of PrEP availability, FSW may face increasing challenges in negotiating condom use within a context of imbalanced power dynamics.^5^ Decreases in condom use, increases in number of partners, or other forms of sexual risk compensation may increase the risk of STI acquisition or attenuate PrEP effectiveness when PrEP adherence is suboptimal.^6^ Therefore, monitoring changes in sexual behavior and condomless sex among FSWs initiating PrEP is needed to assess PrEP delivery programs.

Studies investigating changes in sexual behavior after PrEP initiation have primarily evaluated self-reported measures such as number of partnerships and condom use.^7–11^ However, self-reported sexual behavior measures have significant limitations, such as recall inaccuracy and social desirability bias.^12,13^ Biomarkers of condomless sex such as prostate-specific antigen and Y-chromosomal DNA (Yc-DNA) have been used to assess self-report accuracy as well as to evaluate changes in sexual behavior.^14,15^ The presence of male Yc-DNA in vaginal swabs is a validated biomarker for recent condomless sex that can be detected up to 14 days after semen exposure.^16–20^ A previous study among FSWs in West Africa found that 21% of participants who reported no condomless sex in the last 14 days had detectable Yc-DNA in vaginal swab samples.^21^ However, few studies among FSWs have used objective measures to investigate changes in condomless sex frequency after PrEP initiation.^22^

Sex work has been legal in Senegal since 1969 and is regulated by requiring sex workers to register at a health facility and attend routine checkups.^23^ As part of a PrEP demonstration project in Senegal, we investigated condom use and Yc-DNA detection in the genital tract of sex workers initiating oral PrEP at 4 Ministry of Health FSW clinics in Senegal, West Africa. Our objectives were to assess changes in self-reported sexual behavior and Yc-DNA detection from time since initiation, to evaluate whether self-reported sexual behavioral measures such as recent partners and condom use were associated with vaginal Yc-DNA detection, and to evaluate predictors of Yc-DNA detection.

## MATERIALS AND METHODS

### Study Population

The design, methods, and primary outcomes of the Senegal PrEP Demonstration Project have been described previously.^24^ Briefly, 267 HIV-negative active FSWs (reported paid sex within the past 6 months) medically eligible for PrEP were offered daily oral tenofovir disoproxil fumarate/emtricitabine (300 mg/200 mg once daily; Truvada; Gilead Sciences) at 4 clinics in Senegal and followed up at 1 month, 3 months, and every 3 months thereafter for 1 year. Study visits occurred from 2015 to 2016 and included testing for HIV-1 and HIV-2 and other STIs, medication adherence counseling, condom distribution, risk reduction counseling, clinical examination, and creatinine testing. At each visit, trained staff conducted standardized interviewer-administered questionnaires to assess demographic, clinical, and sexual behavior characteristics among participants continuing to receive PrEP services. Retention in PrEP care was 67% at 12 months after PrEP initiation (Supplemental Digital Content, Table S1, http://links.lww.com/OLQ/A483). No HIV seroconversions were detected over the course of the study among participants retained in care. The study was registered at ClinicalTrials.gov (NCT02474303). All participants in this study provided written informed consent, and the study was approved by the Human Subjects Division Institutional Review Board at the University of Washington, the Institutional Review Board at Westat, and the Senegalese National Ethics Committee for Health Research.

### Self-Reported Sexual Behavior Measures

At initiation and each follow-up visit, participants were asked to report the following measures: number of clients in the last 7 days, frequency of condom use during vaginal or anal sex with clients in the last month (always, almost always, sometimes, almost never, or never), whether the participant has a “main” partner (defined as having regular sexual relations with an individual who did not pay or trade goods or services for sex), number of main partners in the last month, and frequency of condom use during vaginal or anal sex with main partners in the last month (always, almost always, sometimes, almost never, or never). Participants were also asked if they used a condom the last time they had sex with a client and the last time they had sex with a main partner.

### Yc-DNA Detection

Vaginal swabs were self-collected by women at each visit. We used nonproportional quota sampling (stratified on clinic, sex worker registration status, visit number, and retention status) to choose 156 swabs from 121 FSWs for Yc-DNA testing throughout the study period (see Supplemental Digital Content for further details, http://links.lww.com/OLQ/A483). Vaginal swabs were frozen at −80°C and shipped on dry ice in bulk to the University of Washington in Seattle. DNA extraction was conducted using the QIAamp96 DNA blood kit (Qiagen, Hilden, Germany) as described previously.^25^ The Quantifiler Duo DNA Quantification Kit (Applied Biosystems, Waltham, MA) was used for Yc-DNA detection per the manufacturer's instructions as described previously.^20,26^ All Yc-DNA polymerase chain reaction analyses were conducted by female laboratory staff (to prevent contamination with male DNA) who were blinded to study data. We excluded one sample that produced an invalid result (no detection of human autosomal DNA). Two swabs from the same participant at the same visit (one for Yc-DNA detection and one initially designated for STI testing) were inadvertently analyzed and returned discordant results. We excluded the negative result because the likelihood of cross-contamination was low (adjacent samples were negative), the detection level in the positive swab was near the limit of detection, and the assay is highly specific because of low levels of cross-reactivity. Therefore, our sample for our final analysis included 154 swabs from 121 women.

### STI Testing

Limited STI testing was conducted because of logistical constraints such as equipment failure and reagent stockouts and funding (Supplemental Digital Content, Table S2, http://links.lww.com/OLQ/A483). *Neisseria gonorrhoeae* and *Chlamydia trachomatis* nucleic acid amplification testing was performed in Senegal at the Institut de Recherche en Santé de Surveillance Epidemiologique et de Formations using the Roche cobas 4800 System (Roche Molecular Systems, Pleasanton, CA) per the manufacturer's instructions. Syphilis testing was performed by clinic staff using rapid plasma reagin and *Treponema pallidum* hemagglutination assays.

### Statistical Analysis

We summarized demographic and behavioral characteristics of participants using descriptive methods. We dichotomized self-reported condom use into always (consistent) or not always (inconsistent). To investigate changes in sexual risk behaviors over time since PrEP initiation, we calculated the average number of clients reported in the last 7 days and the proportion of participants reporting inconsistent condom use in the last month with main partners and clients by study visit among all women enrolled in the demonstration project. We also calculated the proportion of swabs with detectable Yc-DNA by study visit. We estimated 95% confidence intervals (CIs) for proportions using the Wilson score interval to constrain limits between 0 and 1.^27^ We estimated trends in sexual behavior over time using generalized estimating equations assuming an exchangeable working correlation structure to account for repeated measures on participants.^28^ We used a binomial model with a logit link to model dichotomous outcomes (condom use and Yc-DNA detection) and a Gaussian model with identity link to model the number of clients reported in the last week. All models included month since initiation as a linear predictor and were adjusted for age, education, ethnic group, and clinic site. To evaluate changes in sexual behavior over time since initiation, we tested whether the estimated coefficient on the month since initiation variable was statistically significantly different from 0 (linear models) or 1 (logistic models).

To compare Yc-DNA detection by reported partnerships, we stratified the proportion of swabs with detectable Yc-DNA by reported sex with a client (in the 7 days before swab collection), main partner (in the month before swab collection), or both. To compare Yc-DNA detection by reported condom use, we calculated the proportion of swabs with detectable Yc-DNA by condom use in the last month with clients, main partners, or both, as well as condom use at most recent sex with clients, main partners, or both. We assessed condom use with main partners in the last month only among those reporting at least one main partner in the last month; because the number of clients was ascertained in the prior week, we did not perform a similar restriction for condom use with clients in the last month. We also compared Yc-DNA detection across baseline demographic and socioeconomic measures hypothesized a priori as potential predictors of condomless sex frequency. We estimated odds ratios (ORs) and 95% CIs using generalized estimating equations assuming an exchangeable working correlation structure. All analyses were conducted using R version 3.6.1.

## RESULTS

### Study Population

The demographic and baseline characteristics of the 267 participants enrolled in the PrEP Demonstration Project are shown in Table 1. Sixty-four percent of participants were legally registered sex workers, 41% were of Wolof ethnicity, and 58% reported 2 or fewer clients in the prior week. Participants reported high levels of condom use at initiation (93% and 66% reported always using condoms in the last month with clients and main partners, respectively). Most participants reported feeling “very confident” or “confident” in their ability to use condoms the next time they had sex with clients (90%) and main partners (89%), respectively. Of the 40% of participants who received gonorrhea and chlamydia testing at least once during the study period, 7.5% (8/106) tested positive for gonorrhea and 7.5% (8/106) tested positive for chlamydia. Thirty-four (15.4%) of 221 women tested had a positive *T. pallidum* hemagglutination assay test result at either baseline or at any point throughout the study period. The distribution of demographic and baseline characteristics among the subsample of participants for Yc-DNA testing was similar to that of all the participants in the demonstration project (Supplemental Digital Content, Table S3, http://links.lww.com/OLQ/A483).

**TABLE 1:**
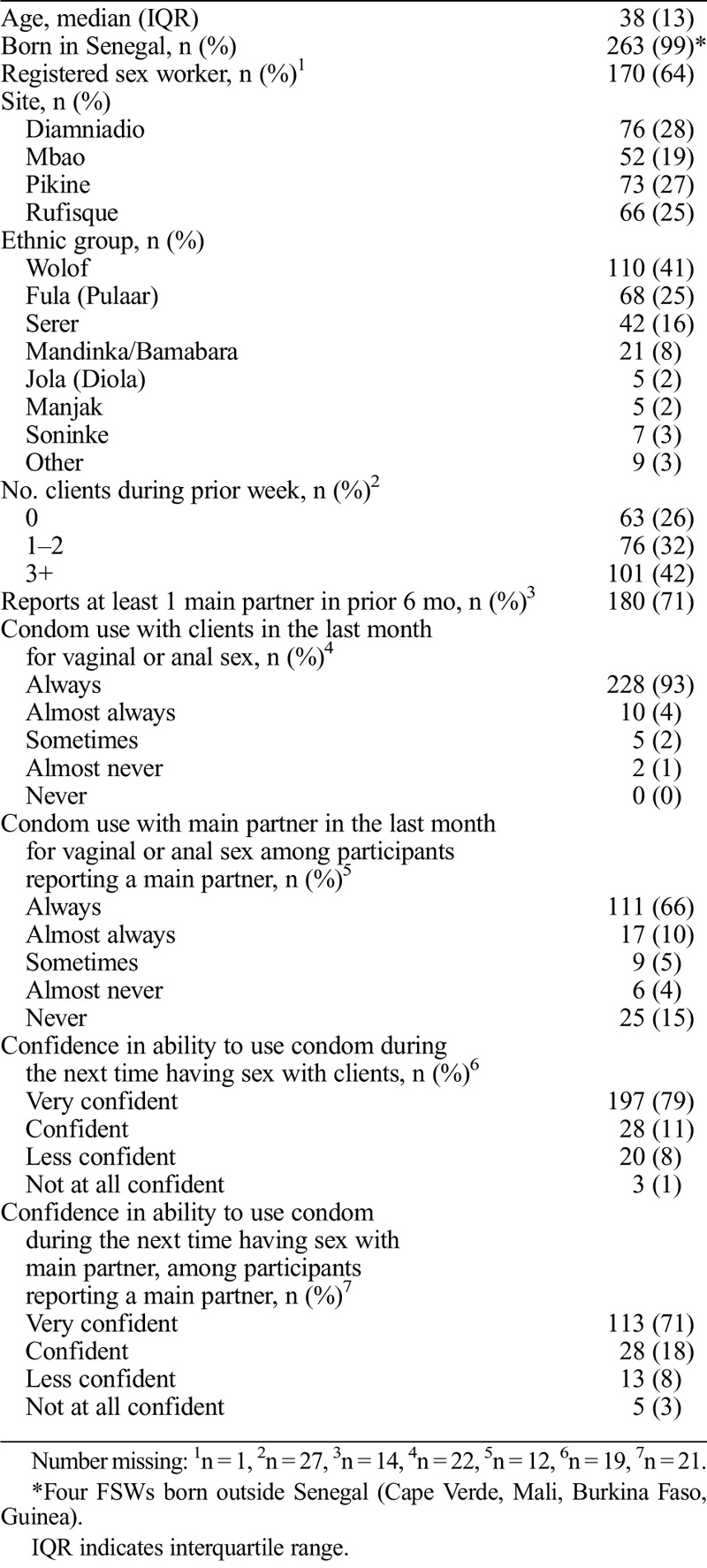
Demographic and Baseline Characteristics of All FSWs Participants Enrolled in PrEP Demonstration Project (n = 267)

### Trends in Sexual Behavior

**Figure 1:**
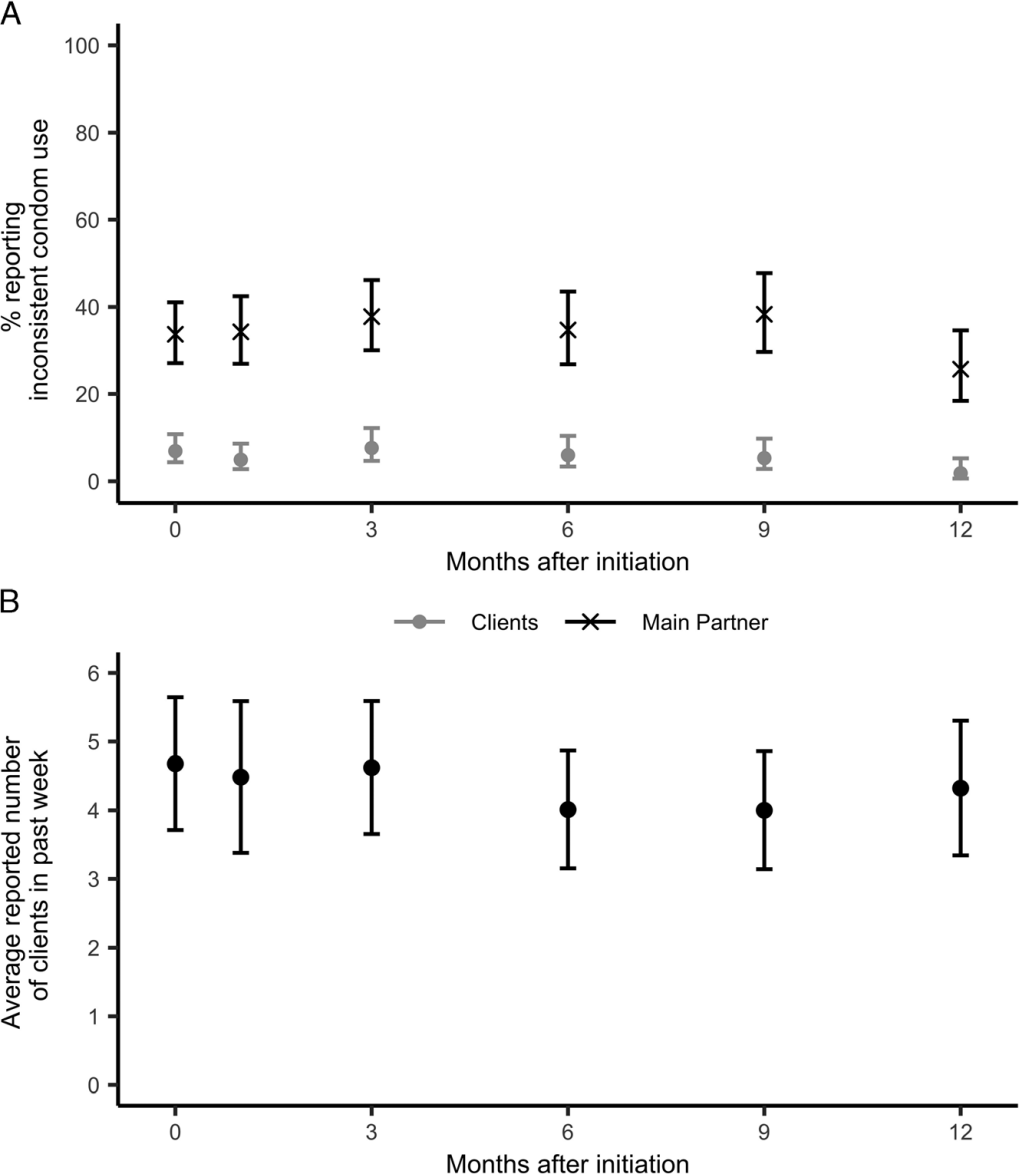
Trends in self-reported sexual behavior among FSWs participating in the Senegal PrEP demonstration project. Error bars represent 95% CIs. Relationship between outcome and month since initiation estimated from adjusted regression models fit using generalized estimating equations assuming an exchangeable working correlation structure. A, Percent of participants reporting inconsistent condom usage in the last month with clients (gray circles) and main partners (black Xs). Inconsistent condom use is defined as not reporting always using condoms. Clients: OR, 0.94 (95% CI, 0.89–1.00; *P* = 0.053); main partner: OR, 0.99 (95% CI, 0.96–1.02; *P* = 0.53). B, Average number of reported clients in the last 7 days among participants. β = −0.04 (95% CI, −0.12 to 0.044; *P* = 0.35).

**Figure 2:**
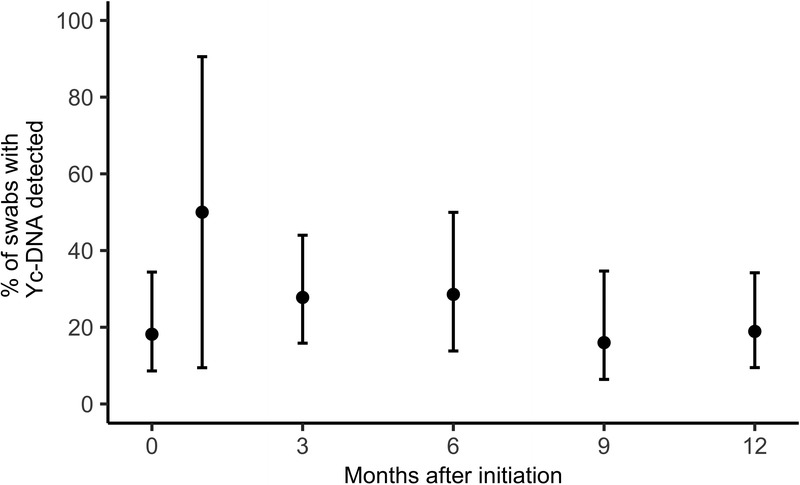
Trends in Yc-DNA detection among FSWs participating in the Senegal PrEP demonstration project. Error bars represent 95% CIs. Number of swabs tested: Initiation: n = 33; month 1: n = 2; month 3: n = 36; month 6: n = 21; month 9: n = 25; month 12: n = 37. Relationship between Yc-DNA detection and month since initiation estimated from adjusted regression models fit using generalized estimating equations assuming an exchangeable working correlation structure. OR, 0.99 (95% CI, 0.90–1.08; *P* = 0.82).

Figure 1 displays trends in self-reported sexual behavior from initiation to study exit at month 12 among all FSW participating in the demonstration project. The proportion of participants reporting inconsistent condom use with clients over the last month decreased from initiation (6.9%; 95% CI, 4.4%–10.8%) to month 12 (1.8%; 95% CI, 0.6%–5.3%). Similarly, inconsistent condom use with main partners also decreased from initiation (33.7%; 95% CI, 27.1%–41.1%) to month 12 (25.7%; 95% CI, 18.4%–34.6%). The average numbers of clients reported in the last week were 4.7 at initiation (95% CI, 3.8–5.6) and 4.3 at month 12 (95% CI, 3.6–5.1). Figure 2 shows the proportion of swabs with Yc-DNA detected by month since initiation. The prevalence of detectable Yc-DNA among tested swabs was similar at initiation (18.2%; 95% CI, 8.6%–34.4%) and at month 12 (18.9%; 95% CI, 9.5%–34.2%).

In adjusted logistic regression models, we estimated the ORs of inconsistent condom use for a 1-month increase in time since PrEP initiation. The odds of inconsistent condom use with clients decreased over time (OR, 0.94; 95% CI, 0.89–1.00; *P* = 0.053), whereas the odds of inconsistent condom use with main partners was stable over time (OR, 0.99; 95% CI, 0.96–1.02; *P* = 0.55). The estimated change in number of reported clients for a 1-month increase in time since PrEP initiation was negative (β = −0.040; 95% CI, −0.12 to 0.044; *P* = 0.35), although this difference was not statistically significant. The median number of clients reported in the past 7 days did not change over time (Supplemental Digital Content, Figure S3, http://links.lww.com/OLQ/A483). We found no evidence of changes in the odds of Yc-DNA detection over time (OR, 0.99; 95% CI, 0.90–1.08; *P* = 0.82).

### Yc-DNA Detection

Thirty-one of 121 women tested had at least one swab with detectable Yc-DNA (25.6%; 95% CI, 18.7%–34.1%), and Yc-DNA was detected in 34 (22.1%) of 154 swabs overall (95% CI, 16.3%–29.3%). Table 2 displays the frequency of Yc-DNA detection stratified by self-reported partnerships and condom use. Yc-DNA detection was more common among participants who reported having had sex with a main partner in the last month compared with those who did not (28% vs. 14%), but the estimated OR was not statistically significantly different from 1 (OR, 2.10; 95% CI, 0.84–5.30; *P* = 0.11). Y-chromosomal DNA detection did not differ by whether participants reported having had sex with a client in the last week (OR, 1.00). Nearly all (96%) of participants reported always using condoms with clients in the month before swab collection, but 22% of samples from these participants had detectable Yc-DNA. Of 58 participants who reported always using condoms with both their main partner and clients in the last month, 15 (26%) had swabs with detectable Yc-DNA. The odds of Yc-DNA detection did not differ significantly by whether or not participants reported inconsistent condom use in the last month with clients (OR, 0.75; 95% CI, 0.10–5.90; *P* = 0.78), main partners (OR, 0.78; 95% CI, 0.25–2.43); *P* = 0.67), or both (OR, 0.80; 95% CI, 0.25–2.55; *P* = 0.71). Results were similar when condom use was assessed at most recent sex rather than over the last month. In addition, none of the other variables we evaluated (age, education, ethnic group, FSW registration status, and site) were statistically significantly associated with Yc-DNA detection in unadjusted analyses (Supplemental Digital Content, Table S5, http://links.lww.com/OLQ/A483).

**TABLE 2:**
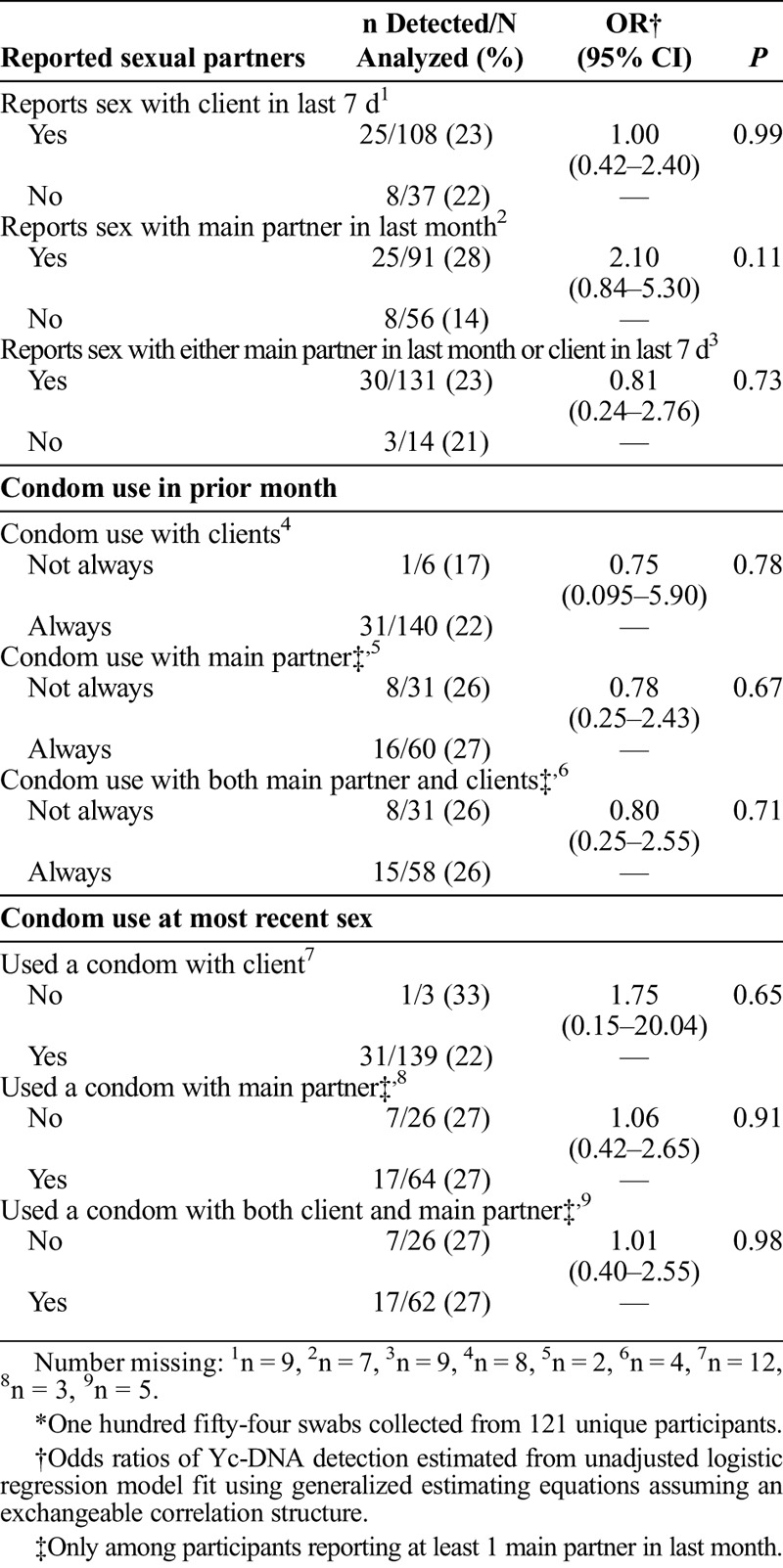
Yc-DNA Detection Stratified by Self-Reported Sexual Partners and Condom Use (n = 154*)

## DISCUSSION

Our objectives were to assess changes in sexual behavior after PrEP initiation, to compare self-reported measures with Yc-DNA detection among FSWs in Senegal, and to assess predictors of Yc-DNA detection. We found no evidence of risk compensation after PrEP initiation as measured by changes in number of clients, condom use, or Yc-DNA detection. Although prior studies in Africa have shown that FSW may charge more for condomless sex, and a discrete choice experiment among FSWs in South Africa predicted that PrEP use would more than double the frequency of condomless sex, our results are consistent with previous studies in PrEP demonstration projects among FSWs in South Africa and Benin that have shown no evidence of risk compensation.^5,8,11,22,29,30^ A recent analysis of a cohort of FSWs initiating PrEP in Benin found no changes in the frequency of prostate-specific antigen or Yc-DNA detection for 24 months since initiation.^22^ Several reasons could explain the lack of risk compensation observed in our study. It is possible that changes in risk behavior may not manifest within the first year of follow-up during PrEP demonstration projects; alternatively, risk reduction counseling and condom provision during PrEP delivery may counteract any perceived economic incentives for engaging in condomless sex. Furthermore, because condoms protect against other STIs and unwanted pregnancy, PrEP use may not have impacted participants' desire to use condoms. Prior research has established a relationship between sexual violence and condomless sex that is partially mediated by difficulties in condom negotiation.^31^ If FSWs have little control over condom use, then PrEP use may have little impact on the frequency of condomless sex. Although difficulty negotiating condom use is an important consideration, study participants reported high confidence in their ability to use condoms with clients and main partners, suggesting that participants in our study had considerable agency in determining their frequency of condomless sex. Because PrEP is rolled out to FSW in resource-limited settings, ongoing assessments of HIV acquisition, STIs, and risk behavior will be needed.

Despite high levels of reported condom use, Yc-DNA was detected in 22% of swabs tested, and self-reported condom use, partnerships, and demographic characteristics did not predict Yc-DNA detection. Prior studies among sex workers in sub-Saharan Africa have not identified consistent predictors of misreporting as compared with prostate-specific antigen detection.^32–34^ In our study, Yc-DNA was detectable in 26% of swabs from participants reporting consistent condom use in the past month with both clients and main partners. This discrepancy may be explained by several factors. Participants may have overreported condom use because of fear of judgment or stigma by health care workers and study staff.^35^ Incorrect condom usage or condom failure could have resulted in unnoticed semen exposure detected by the Yc-DNA assay.^19^ Our measures of reported condom use may not adequately capture nonconsensual condom removal or forced breakage, which have been reported in other settings.^36^ In addition, participants may have misinterpreted the questions or misremembered their condom use. The accuracy of self-reported sexual behavior tends to decrease with longer recall periods or when behavior frequency is high.^13^ These results warrant caution in the use of self-reported sexual behavior for assessing individual-level HIV risk.

Our study builds on prior research of risk compensation by assessing changes in objective biomarker-based measures of sexual behavior over time since PrEP initiation. However, our analysis has several limitations. Our sample size for Yc-DNA analysis was limited by funding and reduced our power to detect predictors of Yc-DNA detection or trends in Yc-detection over time. As condom use was assessed in the month before swab collection, and the sensitivity of Yc-DNA assays is limited beyond 2 weeks after semen exposure, the level of underreporting may be underestimated in our study. Oral sex and/or digital vaginal penetration by clients or main partners may have left traces of Yc-DNA detectable by vaginal swabs, potentially explaining some of the positive Yc-DNA results among women reporting consistent condom use.^17^ We did not assess menstruation or female genital hygiene practices, such as vaginal washing or douching, that potentially could have affected assay performance. Although we ascertained sexual behavior among clients and main partners, we did not ascertain whether participants had other types of partnerships. Our estimate of underreporting may be too high if these partnerships are common and if participants who reported consistent condom use with both clients and main partners would have reported not using a condom with other types of partners. Last, our results may not be generalizable to FSW in other settings or in routine PrEP delivery outside demonstration projects, where factors influencing condom use, partnerships, and sexual behavior reporting could differ. Nevertheless, these results highlight the limitations of using self-reported measures to assess sexual risk behaviors in this population.

In our study of PrEP initiation among FSWs in Senegal, we found no evidence of risk compensation using self-reported condom use, using self-reported number of clients, or by Yc-DNA detection. However, condomless sex as measured by detectable Yc-DNA in vaginal swabs was common among FSWs who self-reported consistent condom use. Studies that rely on accurate measurement of sexual behavior should consider using biomarker measures when feasible. Additional efforts are needed to improve the accuracy of self-reported sexual behavior data.

## Supplementary Material

SUPPLEMENTARY MATERIAL
