# Supplementary material for: Trends in Reported Sexual Behavior and Y-Chromosomal DNA Detection Among Female Sex Workers in the Senegal Preexposure Prophylaxis Demonstration Project
Source: Sex Transm Dis. 2020 Mar 16;47(5):314–20. doi: 10.1097/OLQ.0000000000001175 (PMC7213512; doi:10.1097/OLQ.0000000000001175)
Supplement: SUPPLEMENTARY MATERIAL [file olq-47-314-s001.docx]

**SUPPLEMENTAL DIGITAL CONTENT**

**Table S1: Number of women initiating PrEP and completing follow-up visits**

| **Visit** | **N** | **%** |
| --- | --- | --- |
| Initiation | 267 | 100 |
| M1 | 225 | 84 |
| M3 | 207 | 78 |
| M6 | 188 | 70 |
| M9 | 181 | 68 |
| M12 | 179 | 67 |

**Sampling strategy for Y-chromosomal DNA swabs**

Due to funding constraints, we were only able to select a limited number of swabs for analysis. To ensure adequate sample sizes by site, time since PrEP initiation, and sex worker registration status, we iteratively selected participant ID numbers and ascertained sample availability at each site. We used non-proportional quota sampling instead of random sampling due to small numbers in each stratum and unknown sample availability at each site at the time of sample selection. Samples were selected without knowledge of any characteristics (e.g., reported sexual behavior) other than those identified above. Of the 154 swabs analyzed, 92 (60%) of swabs were from registered sex workers while 62 (40%) were from unregistered sex workers. Among the 121 unique women in our Yc-DNA sample, 89 (74%) contributed one swab, 31 (26%) contributed two swabs, and one (1%) contributed three swabs. The distribution of Yc-DNA samples across site and visit number is shown in Figure S1.

**Figure S1:** Distribution of Y-chromosomal DNA swab samples across site and visit number.


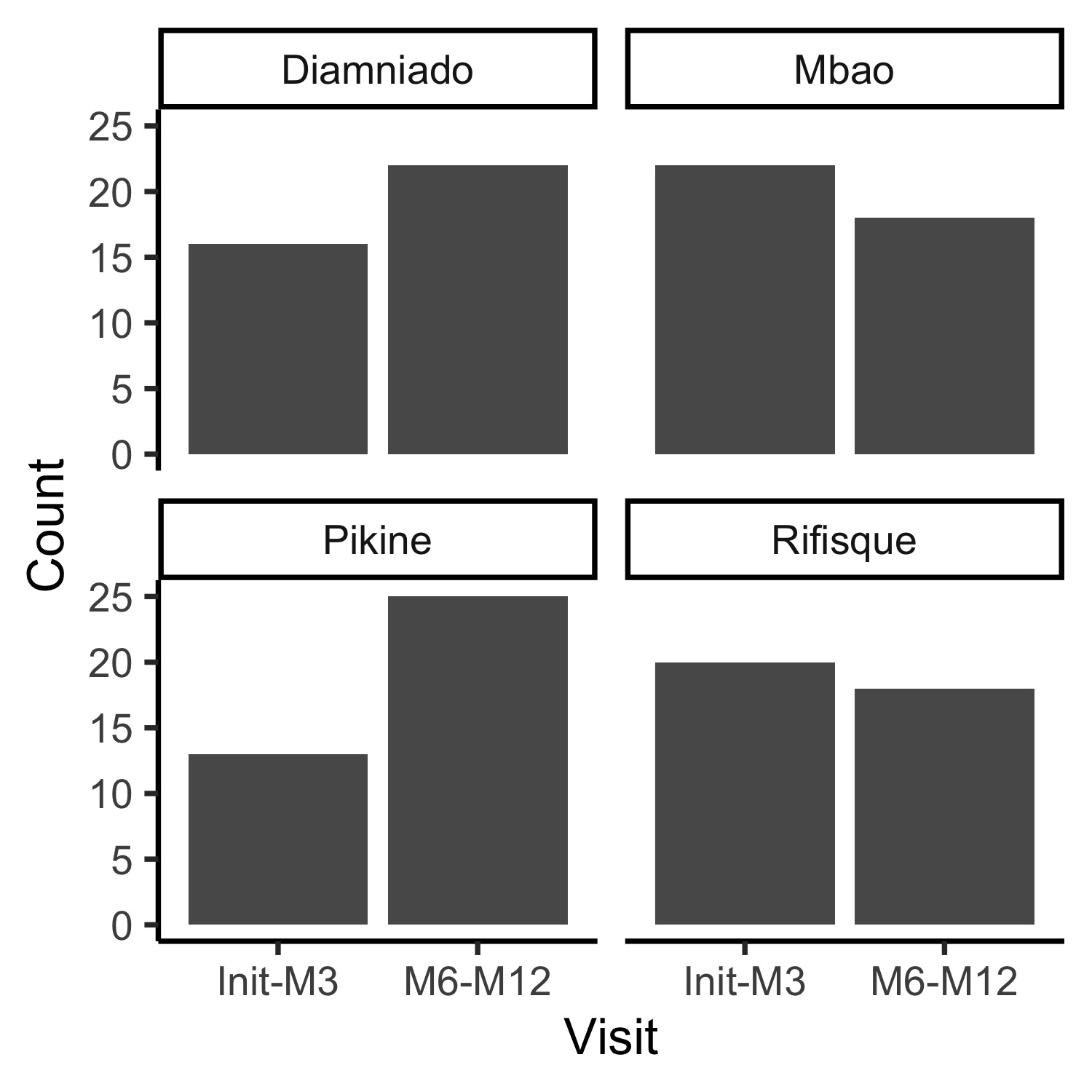


**Table S2: Percent tested for sexually transmitted infections (STI) by study visit.** Limited STI testing was conducted due to logistical constraints such as equipment failure and reagent stockouts and funding.

|  | Percent Tested | | |
| --- | --- | --- | --- |
| Visit Month | Chlamydia | Gonorrhea | Syphilis |
| 0 | 1 | 1 | 20 |
| 1 | 9 | 8 | 39 |
| 3 | 24 | 23 | 46 |
| 6 | 12 | 12 | 23 |
| 9 | 0 | 0 | 29 |
| 12 | 0 | 0 | 39 |

**Figure S2:** Distribution of reported number of clients in the last week and reported number of main partners in the last month across all participant-visits.

*
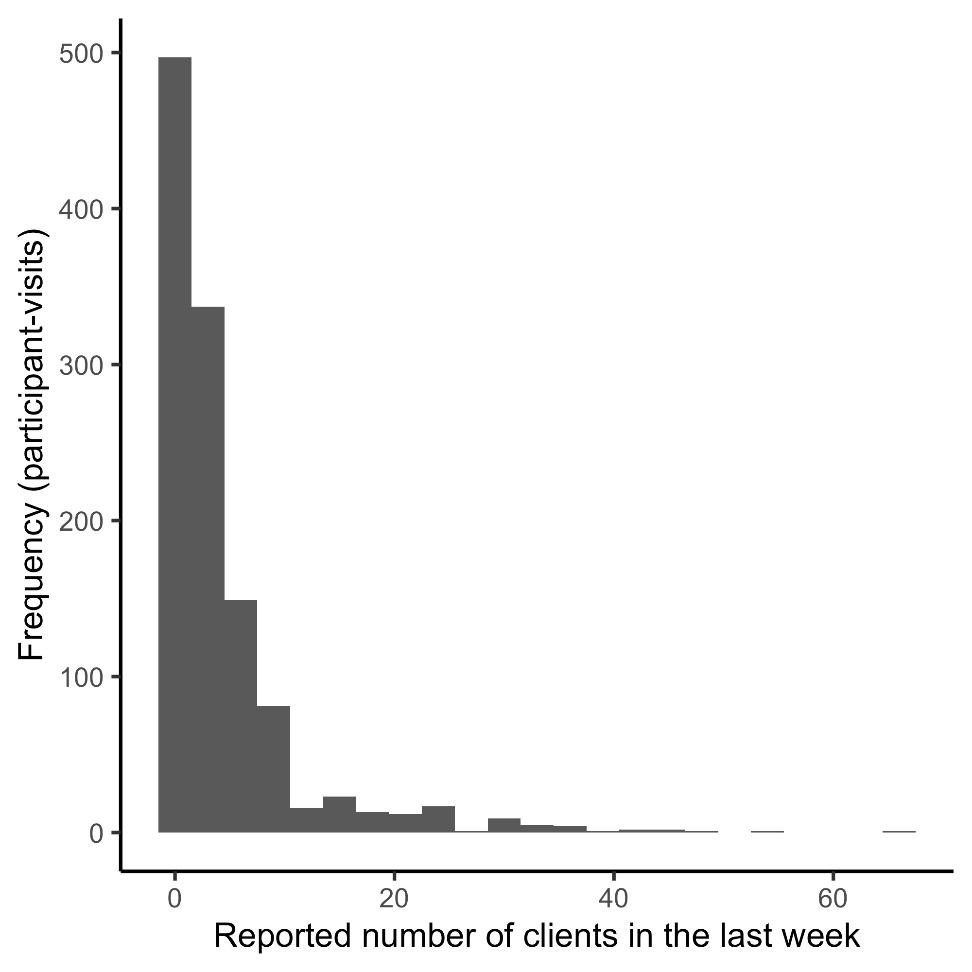

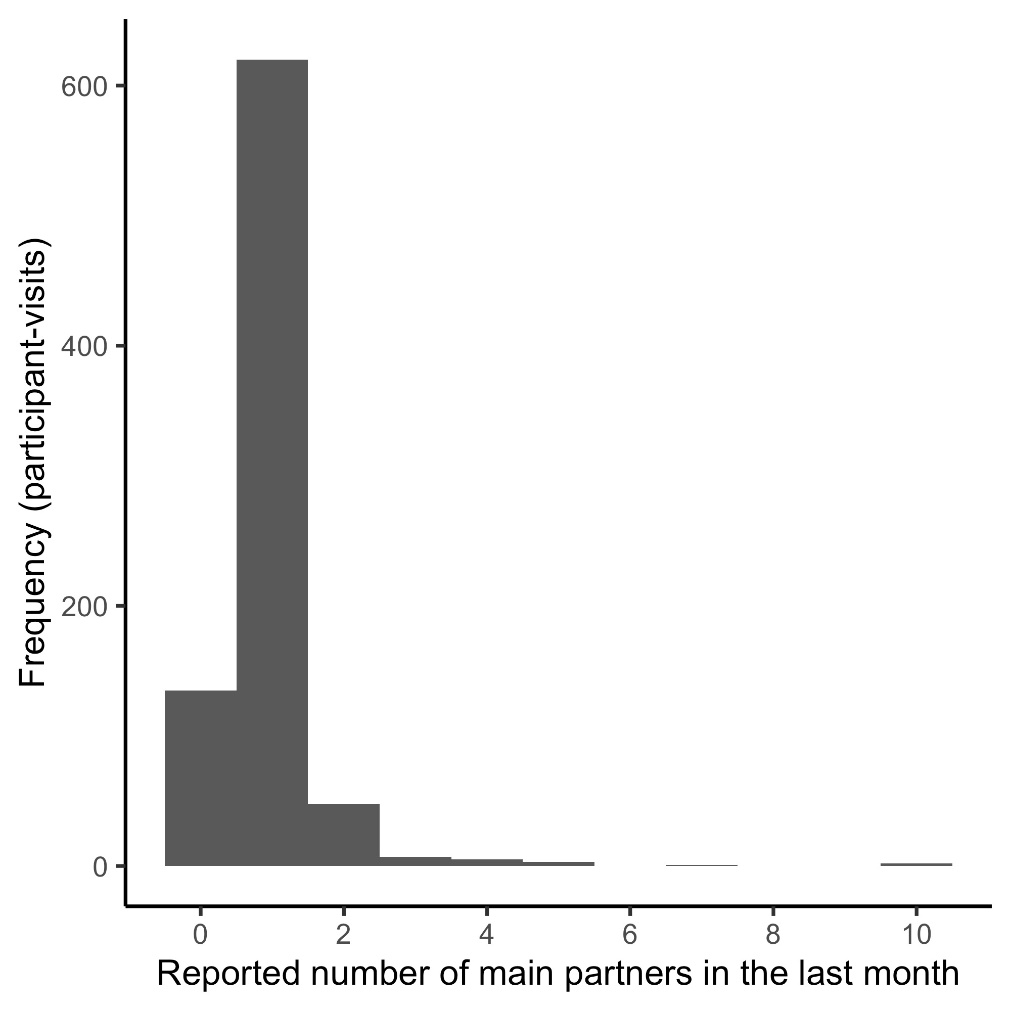
*

**Table S3:** Demographics and characteristics of female sex worker participants in PrEP Demonstration Project with valid Y-chromosomal DNA (Yc-DNA) test results (N = 121)

| **Age** (Median (IQR)) | 40 | (13) |
| --- | --- | --- |
| **Born in Senegal** (%) | 118 | (98%)^*^ |
| **Registered sex worker** (%) | 73 | (60%) |
| **Site** (N (%)) |  |  |
| Diamniadio | 33 | (27%) |
| Mbao | 31 | (26%) |
| Pikine | 30 | (25%) |
| Rufisque | 27 | (22%) |
| **Ethnic Group** (N (%)) |  |  |
| Wolof | 47 | (39%) |
| Fula (Pulaar) | 31 | (26%) |
| Serer | 17 | (14%) |
| Mandinka/Bamabara | 13 | (11%) |
| Jola (Diola) | 3 | (2%) |
| Manjak | 3 | (2%) |
| Soninke | 2 | (2%) |
| Other | 5 | (4%) |
| **Number of clients during prior week** (N (%))^1^ |  |  |
| 0 | 26 | (24%) |
| 1-2 | 39 | (36%) |
| 3+ | 43 | (40%) |
| **Reports at least one main partner in prior six months** (N (%))^2^ | 80 | (69%) |
| **Condom use with clients in the last month for vaginal or anal sex** (N (%))^3^ |  |  |
| Always | 99 | (89%) |
| Almost always | 6 | (5%) |
| Sometimes | 5 | (4%) |
| Almost never | 1 | (1%) |
| Never | 0 | (0%) |
| **Condom use with main partner in the last month for vaginal or anal sex among participants reporting a main partner** (N (%))^4^ |  |  |
| Always | 48 | (66%) |
| Almost always | 8 | (11%) |
| Sometimes | 6 | (8%) |
| Almost never | 2 | (3%) |
| Never | 9 | (12%) |
| **Confidence in ability to use condom during the next time having sex with clients** (N (%))^5^ |  |  |
| Very confident | 53 | (72%) |
| Confident | 15 | (20%) |
| Less confident | 5 | (7%) |
| Not at all confident | 1 | (1%) |
| **Confidence in ability to use condom during the next time having sex with main partner, among participants reporting a main partner** (N (%))^6^ |  |  |
| Very confident | 88 | (78%) |
| Confident | 17 | (15%) |
| Less confident | 7 | (6%) |
| Not at all confident | 1 | (1%) |

^*^3 participants born outside Senegal (Cape Verde, Mali, Burkina Faso)

Number missing: ^1^n=13, ^2‑^n=5, ^3^n=10, ^4^n=7, ^5^n=6, ^6^n=8

**Table S4:** Sample sizes and percentages for underlying data displayed in Figure 1.

**(A)** Percent of participants reporting inconsistent condom usage with clients in the last month

|  | N | % reporting inconsistent condom use with clients in last month |
| --- | --- | --- |
| Initiation | 245 | 6.94% |
| M1 | 222 | 4.95% |
| M3 | 196 | 7.65% |
| M6 | 183 | 6.01% |
| M9 | 169 | 5.33% |
| M12 | 163 | 1.84% |

**(B)** Percent of participants reporting inconsistent condom usage with main partners in the last month (among those reporting at least one main partner in the last month)

|  | N | % reporting inconsistent condom use with main partners in last month |
| --- | --- | --- |
| Initiation | 172 | 27.0% |
| M1 | 140 | 26.9% |
| M3 | 135 | 30.0% |
| M6 | 121 | 26.8% |
| M9 | 107 | 29.7% |
| M12 | 109 | 18.4% |

**(C)** Average number of reported clients in the last seven days

|  | N | Average number of clients |
| --- | --- | --- |
| Initiation | 240 | 4.68 |
| M1 | 221 | 4.48 |
| M3 | 196 | 4.62 |
| M6 | 184 | 4.01 |
| M9 | 167 | 4.00 |
| M12 | 164 | 4.32 |

**Figure S3:** Median number of clients reported in the past week, by month after PrEP initiation.

*
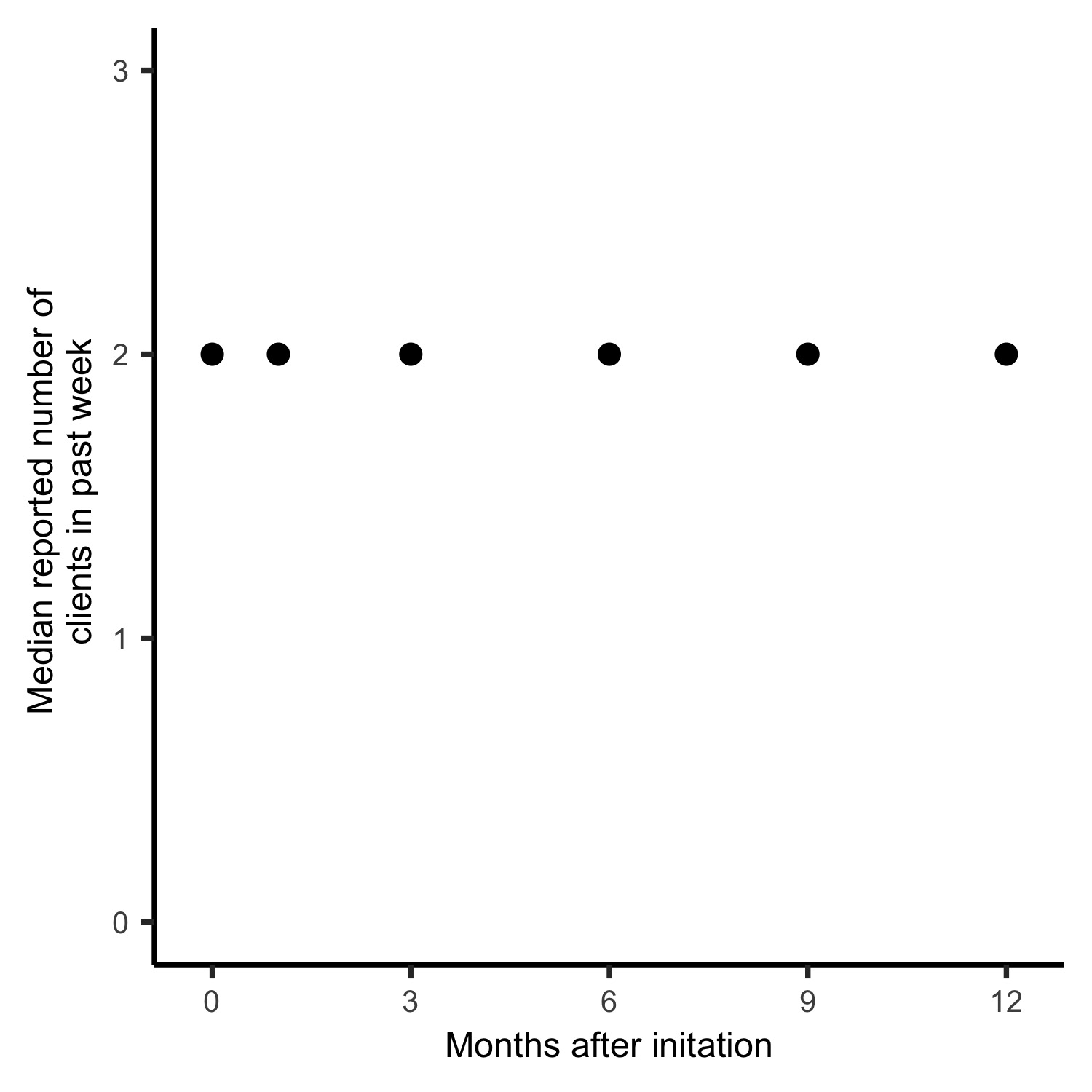
*

**Table S5:** Predictors of Y-chromosomal DNA detection. Unadjusted odds ratios were calculated by logistic regression fit using generalized estimating equations with an exchangeable correlation structure.

|  | **n detected/**  **N analyzed** | **% detected** | **Odds ratio** | **95% Conf. int.** | ***p*-value** |
| --- | --- | --- | --- | --- | --- |
| **Age** |  |  |  |  |  |
| < 30 | 3/21 | 14% | (ref) |  |  |
| 30-39 | 14/44 | 32% | 2.71 | [0.66, 11.17] | 0.17 |
| 40-49 | 14/66 | 21% | 1.56 | [0.39, 6.18] | 0.53 |
| 50+ | 3/23 | 13% | 0.85 | [0.16, 4.62] | 0.85 |
| **Registered FSW** |  |  |  |  |  |
| No | 13/52 | 21% | (ref) |  |  |
| Yes | 21/92 | 23% | 1.04 | [0.46, 2.36] | 0.92 |
| **Ethnic Group** |  |  |  |  |  |
| Wolof | 11/62 | 18% | (ref) |  |  |
| Fula (Pulaar) | 13/39 | 33% | 2.29 | [0.87,6.03] | 0.09 |
| Serer | 3/22 | 14% | 0.73 | [0.19, 2.86] | 0.66 |
| Mandinka/Bambara | 5/16 | 31% | 2.03 | [0.54, 7.57] | 0.29 |
| Other | 2/15 | 13% | 0.69 | [0.15, 3.25] | 0.64 |
| **Education** |  |  |  |  |  |
| None | 16/66 | 24% | (ref) |  |  |
| Primary | 17/69 | 25% | 0.99 | [0.44, 2.22] | 0.97 |
| Secondary | 1/19 | 5% | 0.19 | [0.02, 1.51] | 0.12 |
| **Site** |  |  |  |  |  |
| Diamniado | 9/38 | 24% | (ref) |  |  |
| Mbao | 15/40 | 38% | 1.94 | [0.69, 5.48] | 0.21 |
| Pikine | 3/38 | 8% | 0.26 | [0.06, 1.07] | 0.06 |
| Rufisque | 7/38 | 18% | 0.69 | [0.21, 2.30] | 0.55 |
